# Body mass index in Saudi Arabian children and adolescents: a national reference and comparison with international standards

**DOI:** 10.4103/0256-4947.55162

**Published:** 2009

**Authors:** Abdullah S. Al Herbish, Mohammed I. El Mouzan, Abdullah A. Al Salloum, Mansour M. Al Qureshi, Ahmed A. Al Omar, Peter J. Foster, Tatjana Kecojevic

**Affiliations:** a From the Department of Pediatrics, King Saud University, Riyadh, Saudi Arabia; b From the Ministry of Health, Riyadh, Saudi Arabia; c From the School of Mathematics, Manchester University, United Kingdom

## Abstract

**BACKGROUND AND OBJECTIVES:**

Because there are no reference standards for body mass index (BMI) in Saudi children, we established BMI reference percentiles for normal Saudi Arabian children and adolescents and compared them with international standards.

**SUBJECTS AND METHODS:**

Data from a stratified multistage probability sample were collected from the 13 health regions in Saudi Arabia, as part of a nationwide health profile survey of Saudi Arabian children and adolescents conducted to establish normal physical growth references. Selected households were visited by a trained team. Weight and length/height were measured and recorded following the WHO recommended procedures using the same equipment, which were subjected to both calibration and intra/interobserver variations.

**RESULTS:**

Survey of 11 874 eligible households yielded 35 275 full-term and healthy children and adolescents who were subjected to anthropometric measurements. Four BMI curves were produced, from birth to 36 months and 2 to 19 years for girls and boys. The 3^rd^, 5^th^, 10^th^, 25^th^, 50^th^, 75^th^, 85^th^, 90^th^, 95^th^, and 97^th^ percentiles were produced and compared with the WHO and CDC BMI charts. In the higher percentiles, the Saudi children differed from Western counterparts, indicating that Saudi children have equal or higher BMIs.

**CONCLUSION:**

The BMI curves reflect statistically representative BMI values for Saudi Arabian children and adolescents.

Body mass index (BMI), also known as the Quetelet or Kaup Index, is derived from the equation: Body weight in kilograms divided by length or height in squared meters.^1^,^2^ It is a useful index for measurement of optimal physical growth in children. BMI norms are well established in the World Health Organization (WHO) and the Center for Disease Control (CDC) growth curves.^3^,^4^ These norms have also been established in various countries showing an important ethnic and secular trend effect.^5^ Several attempts have been made to establish BMI curves for Saudi children and adolescents but none were thorough and representative.^6^–^9^ In this study, we present reference BMI curves for normal Saudi children and adolescents and compare it to the WHO and the CDC curves.

## SUBJECTS AND METHODS

A nationwide project to establish normal anthropometric measures for Saudi Arabian children and adolescents was approved and granted by King Abdulaziz City for Science and Technology (KACST), Riyadh, Saudi Arabia. The project was then implemented by cooperative work between the College of Medicine, King Saud University and the Ministry of Health, Riyadh, Saudi Arabia. The guidelines and criteria of Waterlow et al for such projects were followed.^10^ These guidelines require that measurements be done on a well-nourished healthy population, that the sample should include 200 individuals in each age and sex group, the study should be cross-sectional, the sampling procedure should be defined and reproducible, and the measurement should be carefully made and recorded by observers trained in anthropometric techniques using a well-tested design and calibrated equipment at frequent intervals. Sample selection was based on the most recent population census at the start of the study with the assistance of the general directorate of statistics, Ministry of Planning in Saudi Arabia.^11^

Taking an average and perhaps a conservative Saudi family size of 3 children, 14000 households will theoretically yield a sample size of 42000 children and adolescents (0-19 years of age). This was enough to produce a valid and representative result with a standard error of less than 5%. Households were randomly selected using a stratified multistage probability sampling procedure. The details of the selected households (location, name of the family head, and other information) were provided by the above mentioned general directorate of statistics.^11^ As an important prerequisite for this project, a pilot study was performed in Buraidah City targeting 70 households. This succeeded in testing the procedure starting from training teams to locating the households to collection of the data. Minor modifications were done in the procedure and some difficulties and unexpected obstacles were overcome. The questionnaire in its final version contained three major parts: first, the demographic data of all family members and their medical history; second, physical examination; and third, the measurements after assuring eligibility criteria comprised the following: Saudi family with children and/or adolescents, born at term and free of chronic illnesses based on the history and the physical examination.

At least two investigators actively participated in training the teams in the 13 health directorate all over Saudi Arabia. These investigators remained in constant communication with the team heads to solve any unexpected difficulties. Furthermore, they were responsible for reviewing all completed questionnaires sent to the project headquarters in Riyadh before data entry was initiated. The techniques of measurement were done as per WHO recommendations.^12^ This included measuring the child's length (for <2 years old) with two observers with the child in supine position on a flat surface stadiometer with a fixed head and sliding feet ends, or standing height (for >2 years old) performed by one observer following the recommended standards. Length and height measurement was recorded to the nearest 0.1 cm. The weight was performed with minimal clothing and recorded to the nearest 0.1 kg. The equipment was standardized in that two to three new sets were purchased specifically for the project per directorate. This equipment was easily calibrated, repaired, carried, and moved around. Reliability and accuracy was further assured by following the intra- and inter-observer variations done randomly in 1% of the sample. This was randomly performed on the 1^st^ and the 15^th^ household, respectively, by doing the measurement twice. All data were double-checked, multiple frequency tables were scrutinized, and statistical techniques were used to detect deficiencies and inaccuracies. The date were entered utilizing Epi info 2002 CDC, US, software program published by the CDC.^13^ Birth date which was recorded from the birth certificate or the Saudi national identification card was converted from Hegira to Gregorian and the decimal age was calculated. A published software was used. The lambda, mu, sigma (LMS) method proposed by Cole and Green was utilized for curve construction, smoothing of weight and height and the calculated BMI [wt (kg)÷(L or Ht m^2^)] against age for both boys and girls.^14^

## RESULTS

**Figure 1:**
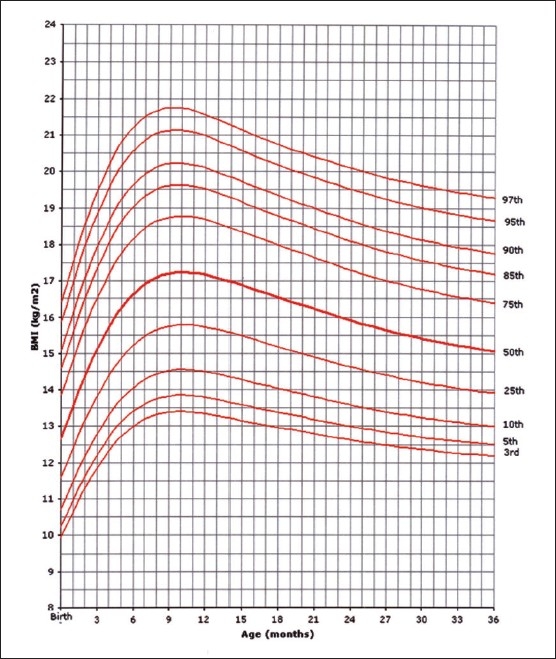
BMI for age-percentiles: girls, birth to 36 months.

**Figure 2:**
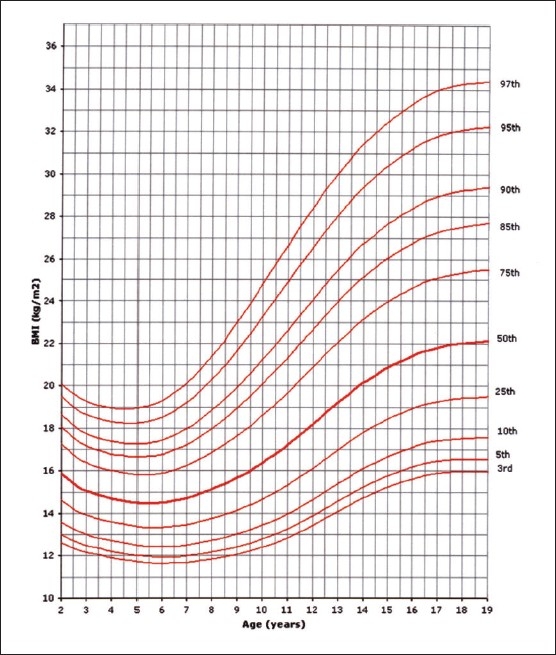
BMI for age-percentiles: girls, 2 to 19 years.

**Figure 3:**
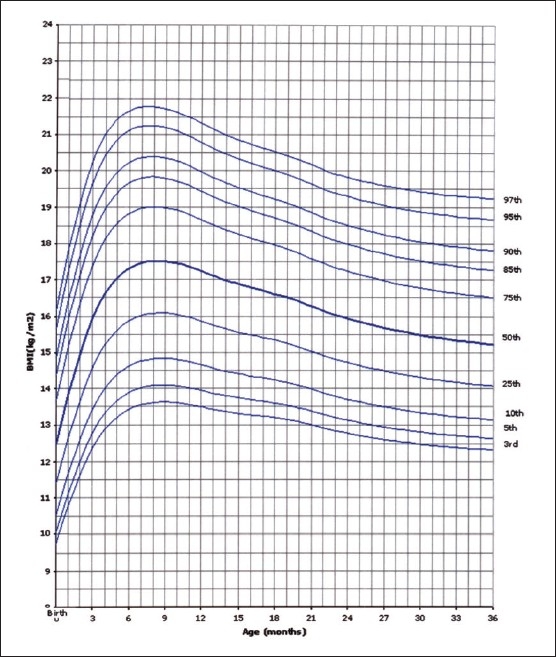
BMI for age-percentiles: boys, birth to 36 months 43×55 mm.

**Figure 4:**
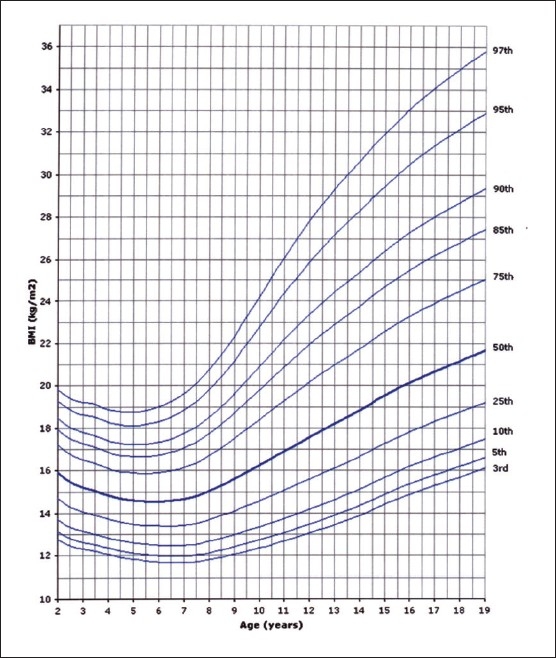
BMI for age-percentiles: boys, 2 to 19 years 43×55 mm.

**Figure 5:**
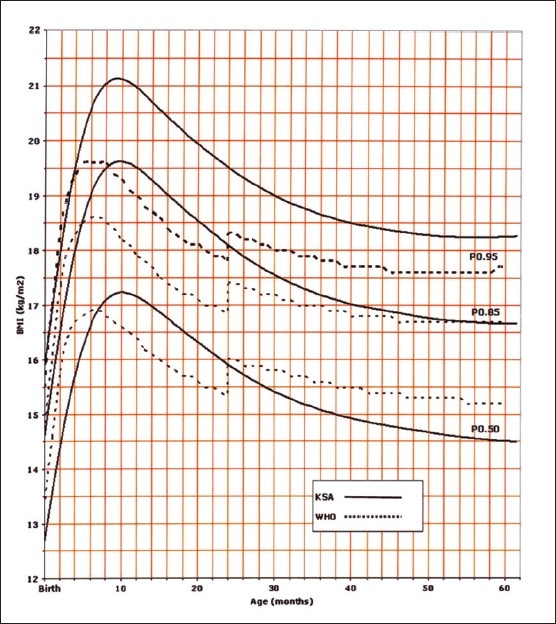
BMI for girls from birth to 60 months: comparison with WHO.

**Figure 6:**
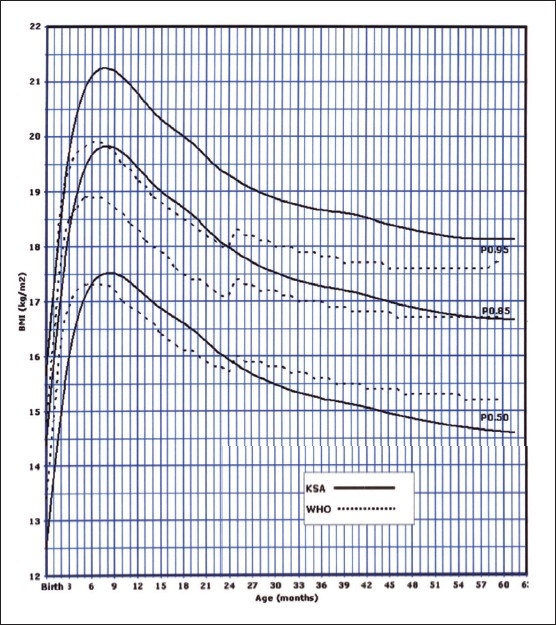
BMI for boys from birth to 60 months: comparison with WHO.

**Figure 7:**
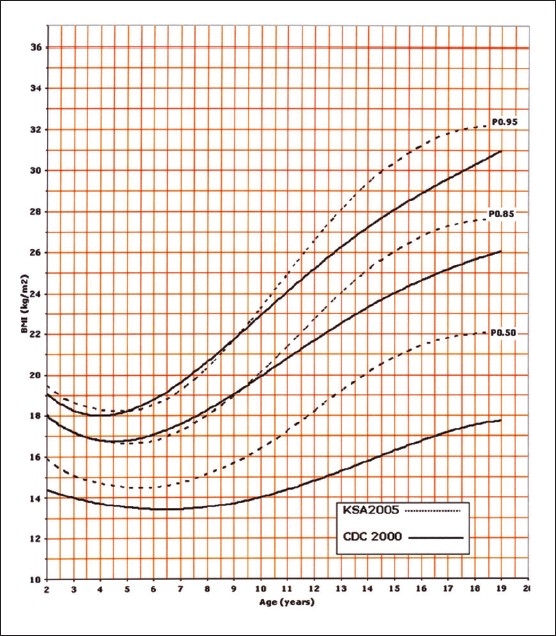
BMI for girls from age 2 to 19 years: comparison with CDC.

**Figure 8:**
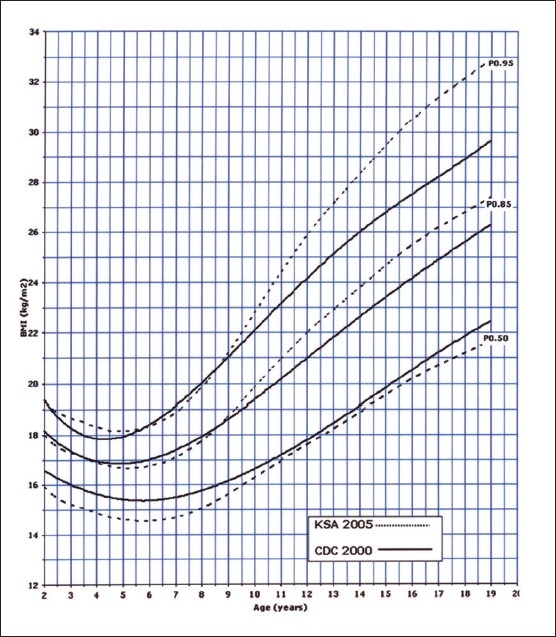
BMI for boys from age 2 to 19 years: comparison with CDC.

Of the 14000 targeted households, 11874 (84.8%) were found eligible as the remaining were either vacant or occupied by families who were non-Saudis or had non-eligible children. However, this number of households yielded 35 279 eligible full-term and healthy children and adolescents for measurements. The sample was well distributed per region indicating good representation (Table 1). The female to male ratio was almost equal (49.5% were females and 50.6% males). Four BMI curves were created: 0-36 month and 2-19 years for both females and males. Ten percentiles were formed similar to the CDC 2000 growth curves. These were the 3^rd^, 5^th^, 10^th^, 25^th^, 50^th^, 75^th^, 85^th^, 90^th^, 95^th^, and 97^th^ percentile. The 85^th^ percentile was included for its important value as a cut-off point for definition of overweight. These curves are shown in Figures 1, 2, 3, and 4. In an attempt to make a comparison between the present study curves and other internationally available curves, we overlapped the present curves with the WHO and the CDC 2000 available curves for the 0-60 months and the 2-19 years of age, respectively (Figures 5, 6, 7, and 8).

**Table 1:** Distribution of eligible children and adolescents for measurements.

Region	Eligible children no. (%)
Makkah Al Mokarramah	6430 (18.1)
Riyadh	6395 (18.0)
Eastern Province	3810 (11.1)
Aseer	3459 (9.9)
Al Jouf	2347 (6.6)
Najran	2241 (6.3)
Al Madina Al Monawarah	2123 (6.0)
Northern Borders	2044 (5.8)
Hail	1945 95.5)
Gizan	1613 (4.7)
Qassim	1293 (3.7)
Al Baha	864 (2.5)
Tabuk	715 (2.0)
TOTAL	35,279 (100)

## DISCUSSION

Establishing norms for every community has become a necessity. Growth charts for Saudi children and adolescents have finally been established for use in the assessment of both healthy and diseased children.^15^ An important parameter of growth is BMI. This has been useful in the assessment of physical growth in children and in the definition of obesity, both in children and adults.^16^ Furthermore, BMI has been found to correlate very well with other long-term health parameters, e.g. blood pressure.^9^,^17^ Among the various tools to assess overweight and obesity, BMI has therefore become more frequently used during the last few decades. BMI is affected by ethnicity.^5^ It is therefore necessary to establish BMI for children and adolescents for the Saudi community where obesity has become prevalent in both children and adults.^18^–^20^ This is due perhaps to changes in the lifestyle of the Saudi community. Weight and BMI have shown a continuous increase over the decades.^5^,^18^ Al Hazzaa demonstrated rising trends in BMI, body fatness, central obesity, and prevalence of obesity among Saudi school boys over the last two decades.^18^,^21^

In establishing any normal value, it is very pertinent to compare these values to what is available internationally. In this study, we have overlapped the calculated BMI for the children and adolescents included in the study with the available WHO (young) and the CDC (old) BMIs. It is very interesting to note that higher percentiles show a difference, illustrating that Saudi children may be equal or even more in BMI compared to their western counterparts. This coincides with the fact that BMIs over the last two decades have been increasing.^21^ This phenomena have also been noticed in other communities such as the US and Australia and some neighboring countries (such as Kuwait and Bahrain).^5^,^22^,^23^ The sex differences noted in the present study depict that female children and adolescents may have higher BMI values compared to males is in line with the reports of Al Shehri et al.^20^ The lower percentiles, however, were less than the WHO and CDC counterparts, particularly beyond six months of age.

We believe that the present study is quite representative for Saudi children and adolescents as it has followed a multi-stage probability random sampling procedure, which is known to be the strongest design to select a representative sample of any population. Furthermore, the methodology of case finding, accurate equipment, and accurate measurements with strong intra- and interobserver variation observations further augment the strength of the representation. Therefore, we highly recommend that these BMI curves be used for reference purposes for Saudi children and adolescents and perhaps for other neighboring countries. These curves should be referred to for assessment of healthy children and children suffering from chronic illness in order to monitor normal growth, an important goal for a proper long-term care and management. Children with specific disorders such as chromosomal anomalies should have their own curves. This has fortunately been established for some children such as Saudi children with trisomy 21.^24^
